# Predictive Accuracy and Densitometric Analysis of Point-of-Care Immunoassay for Adenoviral Conjunctivitis

**DOI:** 10.1167/tvst.10.9.30

**Published:** 2021-08-25

**Authors:** Spencer D. Johnson, Jennifer S. Harthan, Tammy Than, Mary K. Migneco, Ellen Shorter, Meredith M. Whiteside, Christina E. Morettin, Christian K. Olson, Crystal A. Rosemann, Mathew S. Margolis, Leonard W. Haertter, Julia B. Huecker, Bojana Rodic-Polic, Richard S. Buller, Gregory A. Storch, Mae O. Gordon, Andrew T. E. Hartwick

**Affiliations:** 1 Northeastern State University, Tahlequah, OK, USA; 2 Illinois College of Optometry, Chicago, IL, USA; 3 Carl Vinson VA Medical Center, Dublin, GA, USA; 4 Washington University in St. Louis, St. Louis, MO, USA; 5 University of Illinois at Chicago, IL, USA; 6 University of California, Berkeley, CA, USA; 7 Fort Sam Houston, San Antonio, TX, USA; 8 DiaSorin Molecular LLC, Cypress, CA, USA; 9 Ohio State University, Columbus, OH, USA

**Keywords:** conjunctivitis, point-of-care, immunoassay, adenovirus, densitometry

## Abstract

**Purpose:**

Accurate diagnosis of adenoviral conjunctivitis (Ad-Cs) is important for timely and appropriate patient management to reduce disease transmission. This study assessed the diagnostic accuracy of a commercially available point-of-care adenovirus immunoassay and determined whether its predictive accuracy is influenced by signal intensities of test result bands.

**Methods:**

Point-of-care immunoassay (AdenoPlus) testing and quantitative polymerase chain reaction (qPCR) testing was performed on conjunctival swab samples obtained from eyes of 186 eligible adult participants with presumed infectious conjunctivitis and symptoms of ≤4 days. Masked observers assessed signal intensities of the immunoassay test and control bands using densitometry.

**Results:**

Ad-Cs was confirmed by qPCR in 28 of the 56 eyes that tested positive on the AdenoPlus, a 50% positive predictive value (95% confidence interval [CI] = 36.9, 63.1). No adenovirus was detected by qPCR in 128 of 130 eyes that tested negative on AdenoPlus, a 98.5% negative predictive value (CI = 96.3, 100). Sensitivity and specificity were 93% (CI = 84.4, 100) and 82% (CI = 76.0, 88.1), respectively. Viral titers significantly correlated with ratio of test band signal intensities (R^2^ = 0.32, *P* = 0.002). Higher positive predictive value was associated with higher densitometry ratios (receiver operating characteristic [ROC] area = 0.71; 95% CI = 0.59, 0.83).

**Conclusions:**

Densitometric analyses suggest that the diagnostic accuracy of AdenoPlus is influenced by the signal intensity of the test result bands. Visual comparison of the test band intensities by clinicians could reduce the false positive rate of point-of-care immunoassays and aid in the diagnosis of viral infections.

**Translational Relevance:**

Ratiometric densitometry of point-of-care immunoassays could aid clinicians’ decision making in diagnosing infectious diseases, including Ad-Cs.

## Introduction

Infectious conjunctivitis is a prevalent ocular condition, making up as many as 2% of general practice consultations.^1^^–^^3^ Viruses, particularly adenoviruses, are the causative agent in a significant proportion of conjunctivitis cases,^4^^–^^7^ and there is currently no US Food and Drug Administration (FDA)-approved treatment for these ocular infections.^8^^,^^9^ However, unnecessary or inappropriate prescribing of antibiotics for conjunctivitis is commonplace,^10^^,^^11^ and partly attributable to the clinical difficulty in discriminating between viral and bacterial etiologies. In accord, a meta-analysis concluded that clinicians cannot reliably differentiate between bacterial and viral conjunctivitis based on clinical signs and symptoms.^12^

Rapid antibody tests using lateral flow immunoassays for antigen detection are available to aid clinical diagnoses of viral infections.^13^ These tests typically yield a binary “yes/no” result for virus antigen presence within 15 minutes. The AdenoPlus is one such immunoassay, using monoclonal antibodies raised against a conserved adenoviral hexon protein.^14^ It is the only FDA-approved point-of-care diagnostic test for adenoviral conjunctivitis (Ad-Cs).

Diagnostic accuracy is important for the initiation of timely and appropriate clinical management to reduce transmission, duration, and severity of Ad-Cs. An initial study of AdenoPlus demonstrated high positive (94%) and negative (95%) predictive values,^15^ although subsequent studies have reported lower values.^16^^–^^18^ We report a prospective diagnostic accuracy study to determine the predictive accuracy of AdenoPlus using quantitative polymerase chain reaction (qPCR) as comparator. Furthermore, we determined whether test band signal intensities influence the predictive accuracy of this immunoassay.

## Methods

### Study Participants

Institutional review board (IRB) approval was obtained by nine participating clinics and Coordinating Center (Washington University). The study complied with the ethical principles of the Declaration of Helsinki and Good Clinical Practices. Adults (age ≥18 years) presenting with red eye(s) and symptom duration of 4 days or less were invited for eligibility screening. Exclusion criteria included recent ocular surgery, skin vesicles, corneal dendrites, conjunctival membrane or pseudomembrane, corneal infiltrates, corneal ulceration, corneal abrasion, ocular foreign body, or anterior chamber inflammation. All screened participants signed informed consent forms after an explanation of study was provided, and completed a standardized eye examination that included AdenoPlus testing. The study reported here was nested within a National Institutes of Health (NIH)-funded multicenter clinical treatment trial. Participants with positive AdenoPlus test results were randomized to treatment in that trial, with conjunctival swab samples being taken at initial and subsequent follow-up visits to measure viral load over time.

### AdenoPlus Immunoassay and qPCR of Conjunctival Samples

The point-of-care immunoassay test is referred to here as the AdenoPlus, which was manufactured and distributed by Rapid Pathogen Screening (Sarasota, FL) through May 2017 when it was acquired and distributed by Quidel Corporation (San Diego, CA). The test was recently renamed the QuickVue Adenoviral Conjunctivitis Test. Participant enrollment occurred over a nearly 3-year span, and AdenoPlus test kits from multiple lots (lot numbers recorded) were used during study. Sample tests from different lots were routinely assessed to ensure visible red test lines were obtained after exposure to adenoviral antigen (positive control) obtained from Rapid Pathogen Screening.

If both eyes were affected, the first eye affected was selected as the study eye. If both eyes became symptomatic on the same day, the study eye was randomly selected. Study eyes were anesthetized with one drop of proparacaine 0.5% (Valeant Pharmaceuticals; Bridgewater, NJ) and, after 5 minutes, the AdenoPlus test was performed in accordance with manufacturer's instructions and a prior report.^15^ Licensed clinicians applying the test were trained in the procedure prior to the start of the study through practice sessions and by watching a video demonstration. In brief (details in Supplementary Methods), the collector tip was applied to the inferior palpebral conjunctiva 6–8 times before immersion in provided buffer solution. After 10 minutes, the AdenoPlus result window was examined. Presence of a blue control band indicated test validity; if the blue band was absent, the test was repeated with a new device. As per the manufacturer's directions, clinicians were instructed that the presence of a red band, however faint, indicated a positive test for Ad-Cs. Absence of a detectable red band indicated a negative result.

Inferior palpebral conjunctival swab samples were obtained 5 minutes after AdenoPlus sampling. The swab was placed in Universal Viral Transport medium (Becton, Dickinson and Company) and frozen at −80°C within 4 hours of collection. The samples were shipped on dry ice in batches to the Coordinating Center where they were stored at −80°C prior to molecular analysis.

For the first 27 study participants, conjunctival swabs were obtained only from the subset of 11 eyes testing AdenoPlus-positive. These swabs were immersed in vials containing 3 mL medium and were analyzed by qPCR concurrently. The Data and Safety Monitoring Committee then approved the following modifications to increase rigor of the study protocol: (1) conjunctival swab samples were obtained from all screened participants (irrespective of AdenoPlus results); (2) swabs were immersed in vials containing 1 mL medium; and (3) examining clinicians were asked to photo-document the AdenoPlus results window. Initially, the purpose of the AdenoPlus photographs was to enable study personnel to validate clinical examiners’ decisions on whether the test was positive or negative. After participant recruitment had ended, a more quantitative protocol was developed to enable assessment of the test band signal intensities by masked observers (see next section).

Frozen conjunctival swab samples were thawed and nucleic acids were extracted using the NucliSENS easyMAG system (bioMerieux, Durham, NC). The LIAISON MDX instrument and adenovirus analyte-specific reagents used for real-time qPCR assays were obtained from Diasorin Molecular LLC (Cypress, CA). Primers with fluorescein-labeled probes were used to amplify and detect conserved regions of the adenovirus hexon gene. Standard curves were constructed using the Adenovirus Molecular Control (DiaSorin Molecular LLC) to enable quantification of viral titers. Details regarding the assay, including specificity and sensitivity, are in the Supplementary Methods.

### Densitometry of AdenoPlus Immunoassay Bands

**Figure 1.**
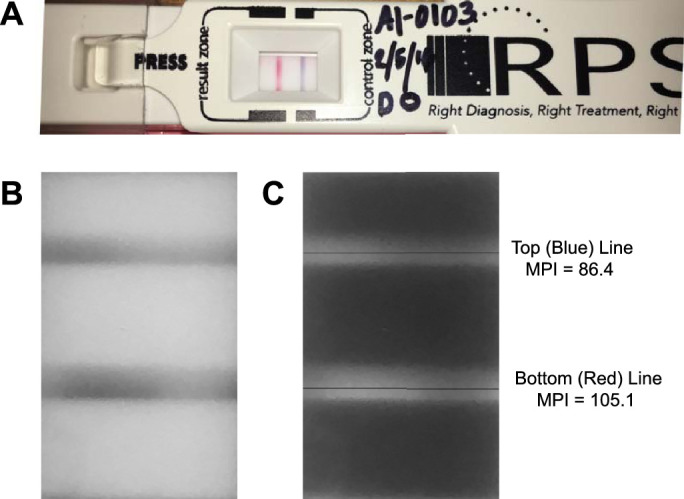
Densitometry of immunoassay bands in AdenoPlus result window. (**A**) Example of valid test (blue band visible) that was deemed positive (red band visible) by the examining clinician. (**B**) Image of the cropped result window that was converted to grayscale. Processed images were always oriented so that bands were horizontal with blue band top-most. (**C**) Image brightness was inverted and maximum mean pixel intensity (MPI) across both bands was determined. The maximum MPI, obtained at locations denoted by thin black line, are shown to the right of the image. The red test-to-blue control band densitometry ratio was calculated to be (105.1/86.4) = 1.22 in this example.

Intensities of AdenoPlus red test bands, relative to blue control bands, were evaluated by densitometry. Examining clinicians photographed the results window of the AdenoPlus, with camera flash on, using their personal mobile devices (Fig. 1A). Digital images were uploaded to a centralized study database.

Images were imported into Photoshop (Adobe, San Jose, CA) and rotated so that the blue control band formed a straight horizontal line in the top half. They were then cropped so only the results window was visible, converted to grayscale mode (color removed; Fig. 1B), and inverted in brightness (Fig. 1C). Each image was standardized to height by width dimensions of 5 inches × 3 inches, with 150 pixels/inch resolution. Contrast and brightness were not adjusted.

Two graders at the Coordinating Center, who were masked to patient data, AdenoPlus results (assessed by examining clinicians), and qPCR values, independently performed the densitometry protocol. Using the “single row marquee” tool, the masked graders placed the pixel-high marquee line so it spanned across the top of the blue control band and moved it down in single-pixel steps until reaching the band bottom, recording the maximum value for mean pixel intensity (MPI). The same procedure was repeated for the red test band if visible. If the red band was not visible, the maximum MPI at locations between 295 and 305 pixels below the blue control band was recorded because the distance between blue and red bands averaged approximately 300 pixels. The MPI across the red test band area was divided by the MPI across the blue control band. Thus, a densitometry ratio of 0.5 signifies that the red test band signal intensity was half as that for the blue control band (see Fig. 1C). This procedure was initially validated in a small pilot study in which the masked observers assessed photographs of AdenoPlus tests that had been treated with adenoviral antigen (positive controls; vials obtained from Rapid Pathogen Screening) or saline (negative controls).

Images flagged by both masked graders as unanalyzable were excluded from further analysis. Flagging reasons included presence of uneven lighting, shadow, or dim/bright overall lighting that affected band visibility.

## Results

### Comparison of AdenoPlus and qPCR Test Results

There were 212 adults (mean age = 34.5 years ± 15.4 years) presenting with presumed conjunctivitis who consented to eligibility screening. AdenoPlus testing and qPCR analyses on conjunctival swab samples were performed on 186 participants (89%; see Supplementary Results for information on others). Thirty percent (56 of 186) of eyes tested AdenoPlus-positive, as judged by the examining clinician, whereas 70% (130 of 186) tested negative. Of eyes testing AdenoPlus-positive, 50% (28 of 56) had qPCR-confirmed adenoviral DNA present in the conjunctival swab samples. For AdenoPlus-negative eyes, 98.5% (128 of 130) were also negative for adenovirus through qPCR testing (Table 1). Using qPCR as the gold standard comparator, the AdenoPlus was determined to have sensitivity of 93.3% and a specificity of 82.1%.

**Table 1.** Number of Eyes Testing Positive (+) or Negative (−) for Adenovirus by AdenoPlus Immunoassay and qPCR

	qPCR +	qPCR −	Total
AdenoPlus +	28	28	56
AdenoPlus −	2	128	130
Total	30	156	186

AdenoPlus parameters: sensitivity = 28/30 = 93.3% (95% CI = 84.4, 100); specificity = 128/156 = 82.1% (CI = 76.0, 88.1); positive predictive value = 28/56 = 50% (CI = 36.9, 63.1); negative predictive value = 128/130 = 98.5% (CI = 96.3, 100).

As this study was part of a randomized treatment trial, follow-up visit data were available for 27 of the 28 eyes that tested positive on AdenoPlus but were negative for adenovirus by qPCR. In all 27 eyes, qPCR results were again negative for adenovirus at the next follow-up visit 1 to 2 days later. These data refute the possibility of spurious qPCR results and confirm that negative qPCR results at baseline were repeatable.

### Densitometry of AdenoPlus Results

Of 186 valid AdenoPlus tests performed, examiners obtained digital images of 142 (76%) tests. Sixteen images were deemed “unanalyzable” by both masked graders and excluded from analysis. In total, the graders calculated red test-to-blue control band densitometry ratios for 126 images with an intraclass correlation of 0.99, indicating high intergrader agreement.

**Figure 2.**
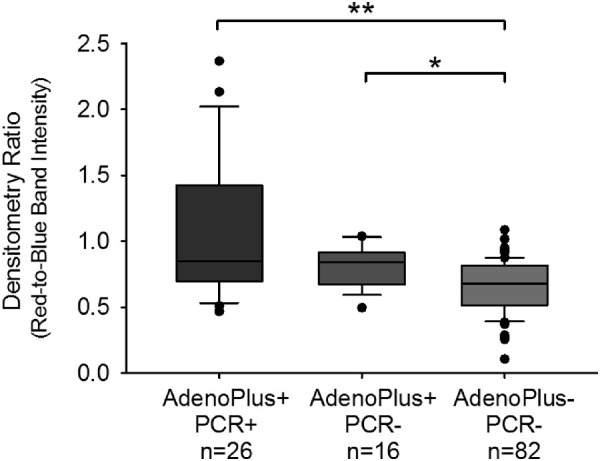
Densitometry ratios (red test band-to-blue control band signal intensities) calculated from images of AdenoPlus test results. Distribution of ratios obtained from positive (+) and negative (−) AdenoPlus tests of eyes testing positive or negative for Ad-Cs by qPCR testing. Lines within each bar represent the median, top, and bottom of the bar represent the 75th and 25th percentiles, respectively. Whiskers show values for 90th and 10th percentiles and dots represent remaining outlier points. *P < 0.05, **P < 0.01, Kruskal-Wallis 1-way ANOVA, Dunn's post hoc.

The median densitometry ratios for the images differed by results on AdenoPlus and qPCR tests (*P* < 0.001, 1-way ANOVA). The median ratio for 26 eyes testing positive on both AdenoPlus and qPCR (0.85, interquartile range [IQR] = 0.73) was higher (*P* < 0.001, Dunn's multiple comparison) than for the 82 eyes (0.68; IQR = 0.30) testing negative on both tests (Fig. 2). In this latter group, the examining clinician determined the red line was absent in the AdenoPlus results window. The objective densitometry ratios therefore supported the examiners’ recorded interpretation of AdenoPlus results.

We sought to address whether discordant results between AdenoPlus and qPCR might be due to examiner misinterpretation of AdenoPlus results. Among eyes classified as AdenoPlus-positive, there were no differences (*P* = 1.00, Dunn's) in median densitometry ratios between qPCR-positive (*n* = 26; median = 0.85, IQR = 0.73) and qPCR-negative eyes (*n* = 16; median = 0.84, IQR = 0.24). Densitometry ratios for AdenoPlus-positive and qPCR-negative eyes were higher (*P* = 0.019) than for eyes testing negative on both tests (Fig. 2). Thus, examiner misclassification of AdenoPlus as an explanation for discordant results was not supported by these data. There were only two eyes with negative AdenoPlus but positive qPCR results (mean densitometry ratio = 0.64), precluding statistical comparisons with other groups.

### Correlation of Densitometry to Adenoviral Titers

**Figure 3.**
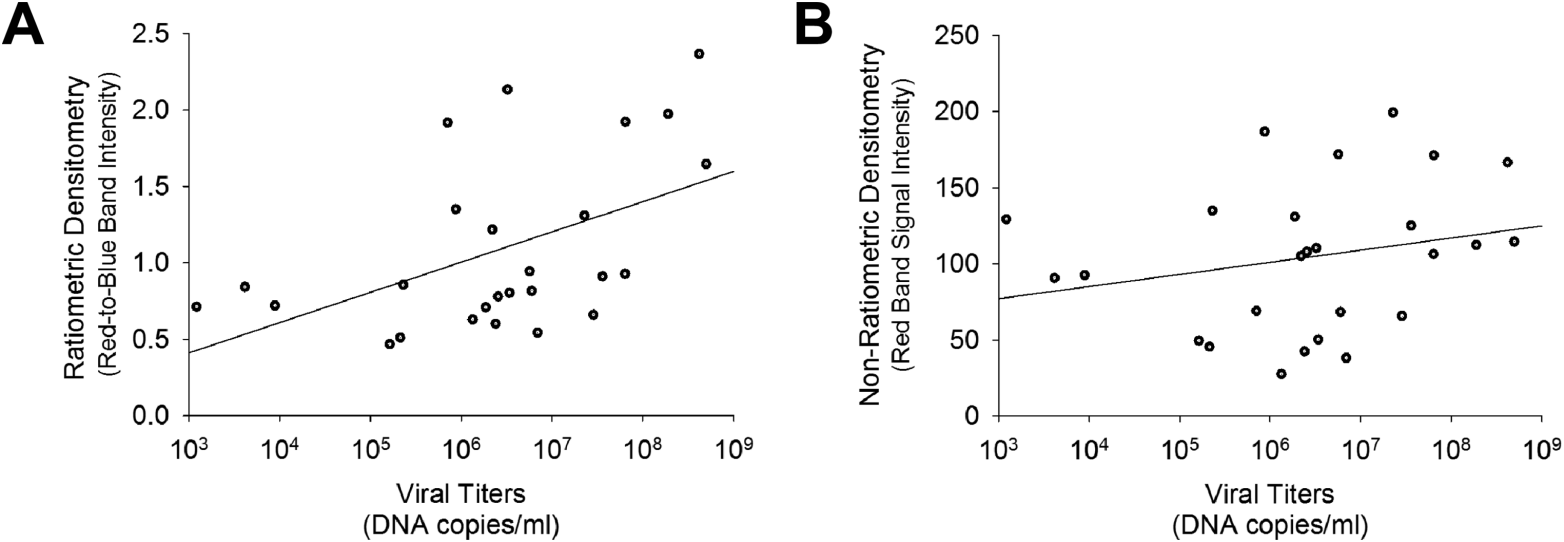
Comparison of densitometry values to quantitative PCR test results. (**A**) The densitometry ratios (red test-to-blue control band signal intensities) were significantly correlated to qPCR-determined adenoviral titers (DNA copies per mL) in the samples that were positive for adenovirus on both AdenoPlus and PCR testing (*n* = 26; Pearson coefficient = 0.57, R^2^ = 0.32, *P* = 0.002). (**B**) Non-ratiometric raw densitometry values (arbitrary units, 8-bit scale) for the red test bands alone did not significantly correlate to adenoviral titers (Pearson coefficient = 0.25, R^2^ = 0.06, *P* = 0.221) in this same subgroup (*n* = 26).

**Figure 4.**
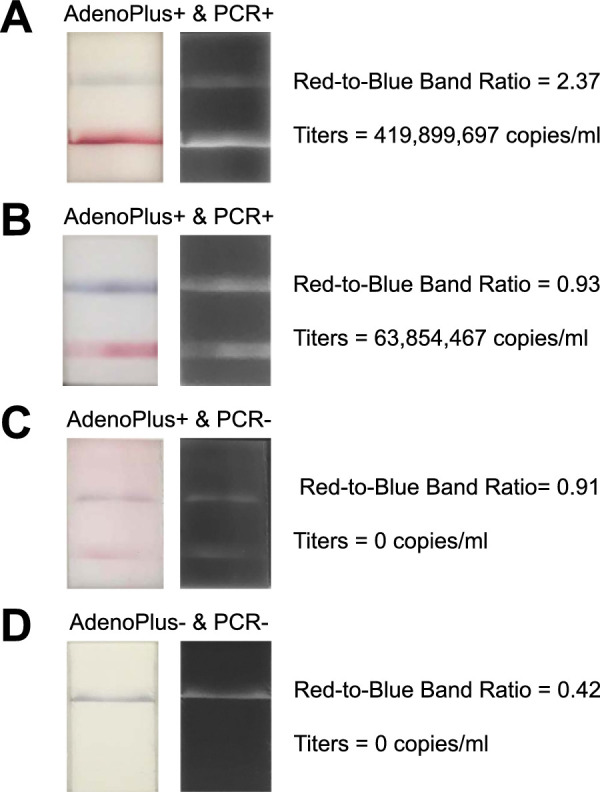
Representative images, with corresponding densitometry ratios (red-to-blue band signal intensities) and adenoviral titers (DNA copies per mL), of AdenoPlus test results from eyes that were (**A, B**) AdenoPlus positive and qPCR positive, (**C**) AdenoPlus positive and qPCR negative, and (**D**) AdenoPlus negative and qPCR negative.

Among eyes testing positive on both AdenoPlus and qPCR tests (*n* = 26; Fig. 3A), higher densitometry ratios correlated with higher adenoviral titers (Pearson coefficient = 0.57, R^2^ = 0.32, *P* = 0.002). This relationship is illustrated by representative images of positive AdenoPlus tests from eyes with different adenoviral titers (Fig. 4). The ratiometric methodology was critical in elucidating the correlation, as the signal intensity of the red bands alone was not significantly (Pearson coefficient = 0.25, R^2^ = 0.06, *P* = 0.221) correlated to adenoviral titers (Fig. 3B).

### Receiver Operating Characteristic Curve

**Figure 5.**
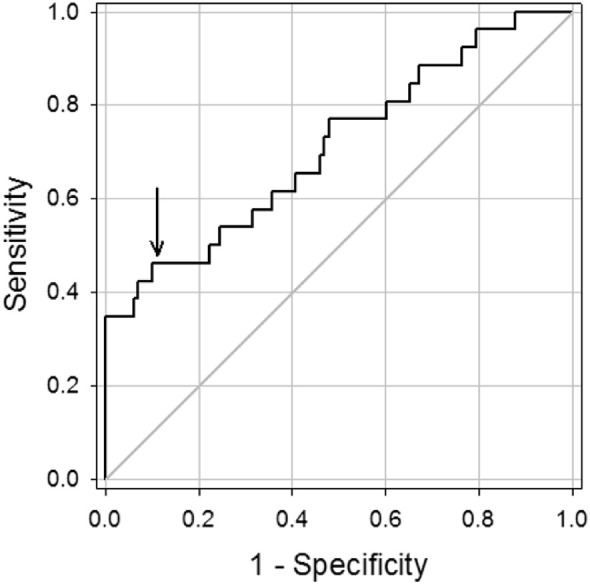
Receiver-operating characteristic (ROC) curve for red test-to-blue control band densitometry ratios for AdenoPlus results in the diagnosis of Ad-Cs (*n* = 124 ratios; *n* = 26 with qPCR-confirmed Ad-Cs). Area under the curve was 0.71 (95% CI = 0.59, 0.83), and was significantly greater than chance with *P* = 0.001. For ratio = 0.9 (denoted by *arrow*), sensitivity = 0.46 (CI = 0.27, 0.67), specificity = 0.89 (CI = 0.81, 0.94) and positive predictive value was 75% (CI = 54, 96).

We explored the diagnostic accuracy for Ad-Cs over the range of AdenoPlus densitometry ratios (Fig. 5). As the densitometry ratio increases (greater red test band intensity compared to the blue control band), AdenoPlus specificity increases, albeit at the expense of reduced sensitivity. When the red test band signal intensity was 90% of that for the blue control band (ratio = 0.9), the specificity and sensitivity of the test was 0.89 and 0.46, respectively, and the positive predictive value increased from 50% to 75% (95% confidence interval [CI] = 54, 96).

## Discussion

The percentage of clinically diagnosed Ad-Cs cases for which adenoviral etiology is confirmed by PCR testing is variable, with estimates ranging from 8 to 82%,^19^^–^^24^ highlighting the challenge of accurately diagnosing Ad-Cs. Although PCR is the gold standard for adenoviral detection,^25^^,^^26^ the technique is costly and time-consuming and usually requires off-site testing. Point-of-care Ad-Cs diagnoses would improve patient management. Timely diagnoses are also important for clinical studies on self-limiting pathologies like Ad-Cs, which typically resolve within 1–2 weeks.^27^

In this prospective multicenter study, the sensitivity of AdenoPlus was 93% and the specificity was 82%, as compared to qPCR testing for adenoviral DNA. However, for clinicians, the more relevant measure of diagnostic accuracy may be positive (proportion of positive tests representing true positives) and negative (proportion of negative tests representing true negatives) predictive values. Nearly all (128/130) AdenoPlus-negative eyes also tested negative by qPCR (98.5% negative predictive value). In contrast, only half (28/56) of AdenoPlus-positive eyes were also positive for adenovirus by qPCR (50% positive predictive value).

Specificity and positive predictive values in this study are considerably lower than those reported in four previous studies (Table 2). Reasons for differences in AdenoPlus performance are not clear. The AdenoPlus exhibited no cross-reactivity with 27 other viral and bacterial pathogens in laboratory testing,^15^ but it is possible that other unidentified pathogens capable of triggering the immunoassay were present in some eyes. Another possibility is that AdenoPlus performance varies depending on adenovirus serotype, although it was reported to exhibit 100% sensitivity in laboratory testing with the 12 most common serotypes.^15^

**Table 2.** Comparison of AdenoPlus Performance Parameters Found in Current Study (RAPID) to Results From Four Prior Studies

	Location	Sample Size	Sensitivity^a^	Specificity^a^	Positive Predictive Value^a^	Negative Predictive Value^a^	% of Sample qPCR+	Age in Years
RAPID	US	186	93%	82%	50%	99%	30/186 (16.1%)	Mean 33.8 range 18–83
Sambursky et al. 2013^15^	US	128	85%	98%	94%	95%	34/125 (27.2%)	Mean 31 range 5–90
Kam et al. 2015^16^	UK	109	40%	96%	85%	71%	43/109 (39.4%)	Mean 40 range 16–85
Holtz et al. 2017^17^	US	46	50%	92%	63%	83%	10/46 (21.7%	Mean 29 range 2–65
^b^Lee et al. 2018^18^	India, US, Sri Lanka, Brazil	500	X	X	78%	X	All: 78% US: 56%	Mean 37 (US) range

a Values based on PCR as the comparator.

b A positive AdenoPlus test was required for inclusion. Values not available for all parameters because PCR was not performed on samples from eyes with negative AdenoPlus tests.

It is also conceivable that differences in AdenoPlus performance could relate to variances in examiner criteria used to define AdenoPlus results. The manufacturer's instructions explicitly direct examiners to classify AdenoPlus test results as positive if the red line is detectable, regardless of faintness. To probe this hypothesis, we developed a protocol for masked densitometric image analysis of AdenoPlus results. Similar densitometry approaches have been applied to molecular biology techniques, such as Western blotting, in quantifying protein accumulation in signal bands.^28^ We found no significant differences in red test-to-blue control band ratios in the two AdenoPlus-positive groups that were either positive or negative for adenovirus by qPCR. Furthermore, the red test-to-blue control band ratios were higher in both AdenoPlus-positive groups compared to tests from eyes with negative AdenoPlus and qPCR results (see Fig. 2). These data indicate that examiner error, meaning AdenoPlus misclassification, was not a major contributor to lower positive predictive values found in this study.

In eyes with qPCR-confirmed Ad-Cs, we found a significant positive correlation between adenoviral titers and the red test-to-blue control band densitometry ratios (see Fig. 3A). Using a criteria ratio of 0.9 (red test band 90% as intense as the blue control band), test specificity and positive predictive value improved (see Fig. 5). We propose the following simplified decision tree for clinicians using the AdenoPlus: (1) if the test is negative (no red band), it is extremely likely that the patient does not have Ad-Cs; (2) if the red test band is approximately the same or greater intensity (>90% as intense) as the blue control band upon visual inspection, it is highly likely that the patient has Ad-Cs; and (3) if the red test band is significantly less intense than the blue test band, a definitive diagnosis cannot be made using AdenoPlus results alone.

Lighting differences between individual images and across different clinics was a study limitation. Overhead lighting could have affected the raw signal intensities for the bands and may have been a contributing factor for the finding that the densitometry of the red test band alone did not correlate to viral titers (see Fig. 3B). However, the ratiometric densitometry method helped to account for differences in lighting during image acquisition, as dimmer images should result in reduced raw signal intensities for both bands, with ratio values remaining relatively stable. The validity of standardizing the red test band intensity as a ratio of the blue control band intensity was confirmed by its correlation with adenoviral titers, whereas the unstandardized red band intensities did not correlate with viral titers (see Figs. 3A vs. 3B). Future studies on the AdenoPlus should use standardized procedures for photograph-documentation. Point-of-care immunoassays can serve as important diagnostic tools for clinicians, and the utility of these tests have gained more public attention during the coronavirus pandemic that began in 2020. This ratiometric densitometry protocol utilized in this study could potentially improve diagnostic accuracy for other point-of-care virus immunoassays, including tests that are currently being used in community screenings for coronavirus disease 2019 (COVID-19).^29^^,^^30^

In summary, we found the AdenoPlus point-of-care immunoassay test to have excellent negative predictive power. This finding has clinical implications, as negative test results can indicate non-adenoviral etiology to the clinician. However, it had a relatively low 50% positive predictive power for Ad-Cs using the manufacturer-recommended criteria of just-detectable red lines to signify positive tests. Our densitometry analyses suggest that visual comparison of the test band intensities by clinicians could improve the diagnostic accuracy of point-of-care immunoassays.

## Supplementary Material

Supplement 1
