# Supplementary material for: Predictive Accuracy and Densitometric Analysis of Point-of-Care Immunoassay for Adenoviral Conjunctivitis
Source: Transl Vis Sci Technol. 2021 Aug 25;10(9):30. doi: 10.1167/tvst.10.9.30 (PMC8399540; doi:10.1167/tvst.10.9.30)
Supplement: Supplement 1 [file tvst-10-9-30_s001.docx]

**SUPPLEMENTAL MATERIAL**

**Methods**

**Conjunctival Sampling for AdenoPlus and qPCR Tests**

The lower lid was pulled down and the AdenoPlus sampling fleece was dabbed on the inferior palpebral conjunctiva and then dragged slightly nasally; this process was repeated 6-8 times, starting temporally and moving nasally across the conjunctiva, with the fleece resting against the nasal palpebral conjunctiva for 5 seconds at the end. The sampling fleece collector was then firmly placed in the test cassette body and the absorbent tip on the cassette body was immersed in the supplied vial of buffer for 20 seconds. The AdenoPlus was laid on a flat surface with the result window facing up, a timer was set, and test results were then read after 10 minutes.

The lower lid was pulled down and a flocked sterile swab applicator (Becton, Dickinson and Company, Sparks, MD) was held approximately parallel to the conjunctival fornix with the handle directed temporally. The swab was placed nasally and dragged temporally along the inferior palpebral conjunctiva. The swab was then rotated 180 degrees, moved back nasally, and held for 5 seconds.

**Quantitative Polymerase Chain Reaction (qPCR) Methodology**

For the DNA extraction, 5 μl of Simplexa™ Extraction and Amplification Control (SEAC) solution (DiaSorin Molecular LLC, Cypress, CA) was added to 200 μl of sample, which consisted of the Universal Viral Transport medium the conjunctival swab was immersed in. The nucleic acids were then isolated by the NucliSENS easyMAG system (bioMerieux, Durham, NC) using the NucliSens magnetic extraction reagents. In brief, the cells were lysed and nucleic acids were bound by magnetic silica particles that were then captured by the magnetic device in the system. The nucleic acids were released from the silica by heating and the purified nucleic acid samples were eluted to a final volume of 50 μl.

The qPCR assays were performed on the LIAISON MDX instrument (DiaSorin Molecular LLC), using the Universal Disc (96 wells) and adenovirus analyte-specific reagents that included a fluorescein (FAM)-labeled integrated probe and 3’ and 5’ adenovirus hexon primers (Diasorin Molecular LLC). The internal control (SEAC) primer pair with a Quasar 670-labeled integrated probe was used to amplify and detect the SEAC DNA fragment.

Each reaction contained 4.0 µl of 2.5 X Universal Master Mix, 0.2 µl adenovirus 3’ hexon primer pair, 0.2 µl adenovirus 5’ hexon primer pair, 0.2 µl internal control (SEAC) primer, 0.4 µl water and 5.0 µl extracted template for a total reaction volume of 10 µl. PCR amplification with real-time detection was performed using the following cycling parameters: denaturation at 97 ◦C for 120 s followed by 40 cycles consisting of denaturation at 97 ◦C for 10 s, anneal/extension at 60 ◦C for 30 s. Fluorescence was measured at end of the each anneal/extension period with quantitative values calculated by LIAISON® MDX Software.

Standard curves were constructed using the Adenovirus Molecular Control (DiaSorin Molecular LLC). Each standard was extracted in a singlicate with each extract amplified in quadruplicate in a single run, and standard curves were used for all subsequent viral load determinations. Positive controls consisting of concentrated adenovirus sample and negative controls of Universal Viral Transport medium alone were included in each sample batch

This analyte specific reagent qPCR assay used in this study is used routinely in clinical laboratories and has been shown to have broad adenovirus genotype coverage [28]. We constructed a standard curve using serial dilutions of the Simplexa™ Adenovirus Molecular Control (DiaSorin Molecular LLC, Cypress, CA). The calibrator values ranged from 200 – 20,000,000 copies/mL, with each concentration tested in four replicates. The lower limit of detection was determined to assess the sensitivity of the assay used in this study. Dilutions of Adenovirus Linearity Panel Standards (Exact Diagnostics, Fort Worth, TX) were tested in 10 - 20 replicates. Based on probit analysis using IBM SPSS Statistics® software, the lower limit of detection, with a 95% confidence interval, was determined to be 182 copies/mL. Specificity of the assay was assessed using samples from clinical patients known to be positive for common viruses, including CMV, EBV, HHV-6, HHV-8, Parvovirus B19, VZV, HIV-1, HCV, HSV-1, HSV-2, and JCV. No cross-reactivity was observed for any of the viruses tested.

**Results**

In total, 212 individuals were screened for the study and both a valid AdenoPlus test and a conjunctival swab sample were obtained from the study eye of 186 of these participants (see results in Table 1). For the remaining 26 individuals, the reason for the incomplete data collection was as follows:

- 4 that were screened but declined study participation prior to both AdenoPlus testing and conjunctival swab sampling
- 16 with negative AdenoPlus tests but had no qPCR test results because they presented before the protocol change requiring conjunctival swabs to be obtained from all eligible eyes (see Methods)
- 2 with negative AdenoPlus test but declined study participation prior to conjunctival swab sampling
- 1 with positive AdenoPlus test but declined study participation prior to conjunctival swab sampling
- 1 with positive AdenoPlus test but was ruled ineligible for treatment trial inclusion (thyroid disease) and no conjunctival swab was obtained
- 2 with invalid AdenoPlus tests (no visible blue line) despite repeated testing
